# Patients' perceptions of the quality of nursing services

**DOI:** 10.1186/s12912-022-00906-1

**Published:** 2022-05-27

**Authors:** Ali Reza Yusefi, Shakiba Rohani Sarvestani, Zahra Kavosi, Jamshid Bahmaei, Morteza Mortazavi Mehrizi, Gholamhossein Mehralian

**Affiliations:** 1 Department of Public Health, School of Health, Jiroft University of Medical Sciences, Jiroft, Iran; 2 Dr. Mir Hospital, Shiraz University of Medical Sciences, Shiraz, Iran; 3 Health Human Resources Research Center, School of Health Management and Information Sciences , Shiraz University of Medical Sciences, Shiraz, Iran; 4 Student Research Committee, School of Management and Information Sciences, Shiraz University of Medical Sciences, Shiraz, Iran; 5 Department of Occupational Health Engineering, School of Public Health, Shahid Sadoughi University of Medical Scince, Yazd, Iran; 6 Pharmacoeconomics and Pharmaceutical Administration, School of Pharmacy, Shahid Beheshti University of Medical Sciences, Tehran, Iran; 7 Nottingham Business School, Nottingham Trent University, Nottingham, UK

**Keywords:** Nursing services, Quality, Patient, Nurse, Hospital, Iran

## Abstract

**Introduction:**

The quality of nursing services is one of the main factors accelerating patients' recovery. The present study aimed to examine patients' perceptions of the quality of nursing services in the teaching hospitals of Iran.

**Methods:**

This cross-sectional research was a descriptive-analytical study conducted in 2021, in which 1067 patients were selected as the research sample. The Qualipak nursing quality questionnaire (QUALPAC) was used to collect the required data. Data were analyzed using t-test, ANOVA, and Pearson correlation coefficient using SPSS software version 23.

**Results:**

From the patients' perspective, the mean and standard deviation of the quality of nursing services was 191.47 ± 19.51. Among the quality dimensions, all services quality: psychosocial (91.34 ± 9.34), physical (65.72 ± 10.18), and communication (34.41 ± 6.21) were placed at the moderate level. A significant association was found between patients’ age and nursing service quality. The perceived nursing service quality was subject to sex (*P* = 0.01, t = 1.921) and place of residence (*P* = 0.02, t = 1.873).

**Conclusion:**

According to the findings, the quality of nurses 'care was "moderate" from the patients' perspectives. Planning is recommended to reinforce and promote the quality of nursing services.

## Background

Quality refers to a complicated structure of values, beliefs, and attitudes in individuals interacting in the health care system. Care is also considered an essential component of health services [1]. In other words, the quality of services is the service’s potential to satisfy the expressed needs [2] and the extent to which the service recipient’s expectations are met [3]. The quality of health services is achieving the most desirable health outcomes [4] so that the services provided are effective, efficient, and economical [5]. One of the main missions of healthcare organizations like hospitals is to provide quality services to meet patient expectations. To this end, the quality philosophy should be first institutionalized within the hospital settings, particularly in the nursing services [6, 7]. This can reduce the length of hospitalization and enhance patients’ satisfaction [8]. In addition, service quality leads to lowering healthcare costs [9]. Hence, given that a large portion of a society usually will be eligible to receive hospital services in different life milestones, it necessitates nurses deliver high-quality services [10]. Nurses form the largest group of staff providing health services [11], and they are the main foundation of the process promoting the quality of care services. Accordingly, their performance plays a crucial role in advancing organizational goals [12]. The professional competence of this occupational group is critical in fulfilling the health system’s mission as such, the level of their professional competence and care is one of the main concerns for health systems and health care providers in different countries [11]. Since patients are most frequently in contact with nurses, some experts exclusively attribute the acceptability of the provided services to nurses, and the prominent role of other treatment groups is often overlooked [13].

All patients have the right to receive high-quality services, and all caring nurses are in charge of facilitating this goal. In most countries, hospitals’ accreditation and rating are affected by nursing care and its quality [14]. Moreover, nurses must be legally and ethically accountable for the quality of the offered care [15]. The quality of nursing care is the nurse's response to patients’ physical, psychological, emotional, social, and spiritual needs so that they can return to their healthy and normal lives while patients and nurses’ have also been satisfied [16]. From another perspective, nursing care is of great importance in health care systems [17], and care is a basic and pivotal reality in nursing [18]. Schroyer et al. highlighted the necessity of quality measurement in a competitive healthcare environment [19]. On the other hand, due to the increase in health care costs, the persistent improvement of the quality of nursing services is necessary, and quality control of nursing services is also a must to promote patient satisfaction [20]. Nantsupawat et al. showed that from the patients' point of view, quality nursing care facilitates access to physical, psychological, and social care [21]. It is well addressed that nursing services can decrease patients’ recovery time and help them go back home [13]. In contrast, low-quality services cause patients to experience severe symptoms, hospital infections, and psychological dysfunction such as anxiety and depression [22].

Given that nurses provide the largest proportion of treatment care services to patients, they play a significant role in improving the quality of services [23]. Patients have the right to receive acceptable and high-quality nursing care [24]. In today’s health systems, the quality of nursing services is exposed to many challenges. Furthermore, some recent studies on nursing have indicated that the quality of nurses' care is relatively low [25, 26]. The results of a study in 2012 in 12 European countries and the United States revealed the low quality of nursing care in countries such as Ireland and Greece [26].

However, most patients and clients demand high-quality services due to their increased awareness in the field of health and also the increased costs of health services [27].

According to some experts, promoting the quality of services enhances productivity, reduces costs, and thus increases patient satisfaction [28]. Evidence suggests that understanding the different perspectives of stakeholders, including patients, caregivers, cost payers, and the general public, on care delivery is of paramount importance to design appropriate programs to improve service quality [29]. On the other hand, evaluating the quality of nursing care make improvements in the way services are provided so that services are offered in accordance with standard health patterns [30]. Relevant studies show that the assessment of the care quality leads to fostering practical skills and promoting competencies, detecting shortcomings, providing more accurate services, eliminating problems and dissatisfaction in departments, thereby ultimately motivating the provision of higher quality care and meeting patients’ needs [31]. Previous studies suggest that continuous and periodic review of the quality of nursing care from different perspectives can facilitate the detection of strengths and weaknesses and contribute to developing optimal programs to improve the quality of services. Neishabory et al. found out that the quality of nursing care was acceptable from the perspective of 92.6% of nurses regarding the psychosocial dimension and 56.8% of nurses regarding the communication dimension. Moreover, the quality of nursing care was acceptable from the perspective of 31.6% of patients regarding the psychosocial dimension and 24.7% of patients regarding communication [32]. In their study, Shannon et al. revealed that the average quality of care was 81.69% for patients, 73.86% for nurses, and 83.55% for physicians [33].

Given that nursing care is one of the most fundamental issues in nursing and results in improving the level of services offered to the community and the more immediate recovery of patients, the present study aimed to assess the quality of nursing care from the perspective of patients in teaching hospitals affiliated with the Shiraz University of Medical Sciences in 2021. Compared to previous research, this study was performed at several centers with a relatively large sample. In addition, we assessed a wide range of patients' views who have been admitted to different wards, leading to a better understanding of the nursing service quality.

## Methods

### Design and setting

This is a descriptive, cross-sectional study conducted in 14 hospital settings where 3043 nurses are working.

### Participants

The study population encompassed patients admitted to these hospitals. Using the Cochran formula and the confidence level as 95%, d (3%), and P 50%, a sample of 1067 was estimated.

$$n=\frac{{z}_{\! {~}^{1-{\alpha \!\!}}\left/\!\!\!{~}_{2}\right.}^{2}P\left(1-P\right)}{{d}^{2}}$$

These numbers were selected using the stratified sampling method from each hospital. In each hospital, patients were randomly selected using the aforementioned method proportionate to the size of each ward. Inclusion criteria were willingness to participate in the study, being aged above 18 years, staying in one of the hospitals for at least 48 h, and not suffering from a mental disorder as confirmed by the treating physician. Exclusion criteria were inability to respond, lack of awareness, and unwillingness to participate in the study.

### Instruments

Patient Demographic Information Questionnaire and QUALPAC (Quality Patient Care Scale [34] were used as data collection tools. QUALPAC contains 72 items examining the quality of nursing services in psychosocial (33 items), physical dimension (26 items), and communication dimension (13 items) dimensions. Each item was scored using a Likert scale with the following options: very low (score 1), low (score 2), medium (score 3), high (score 4), very high (score 5). Regarding the score ranges, the scores of the psychosocial dimension were classified as low (33–77), moderate (78–122), high (123–165). The physical dimension was scored as low (26–60), moderate (61–95), and high (96–130). The communication scores were low (13–30), moderate (31–48), and high (49–65). The total scores of QUALPAC were low (72–168), moderate (169–265), and high (266–360). Haghighi Khoshkho et al. confirmed the validity of this tool and adapted it to Iranian culture [35]. The reliability of the questionnaire is also confirmed in previous studies [36, 37].

### Procedure and statistical analysis

Regarding the research procedures, two of the researchers (SRS and MMM) referred to the concerned hospitals on different weekdays in morning, evening, and night shifts and distributed questionnaires, and collected the required information. Individuals willingly took part in the study and filled out the questionnaire. After obtaining the necessary permits from the Shiraz University of Medical Sciences and explaining the objectives of the project to the participants, the confidentiality of information was emphasized, and their verbal satisfaction was obtained. Questionnaires were then distributed among the patients. Questionnaires were completed by the patients; however, some patients asked the research team (SRS and MMM) to help them fill out the survey.

Then the questionnaires were completed independently and returned. Afterward, the collected data were imported to SPSS software version 23. We performed Pearson’s correlation to test the relationship between the nurses’ services quality and patients' age. T-test has been used to investigate the mean difference between the nurses’ services quality based on patients' sex and place of residence. To analyze if there are any differences between the nurses’ quality services and participants' profiles such as marital status, education, and income level variables, the ANOVA test has been applied.

## Results

According to the descriptive findings, most of the patients were in the age group of 20–35 years (35.80%), male (56.98%), and urban residents (55.58%), married (47.71%), with a diploma and higher education (64.94%) and an income level of 10–20 million Rials (380.735–764.47 US $) (51.45%) (Table 1).

**Table 1:** Frequency distribution of patients

Variables	Category	Frequency (Percent)
**Age (year)**	< 20	129 (12.09)
	20–35	382 (35.80)
	36–50	342 (32.05)
	> 50	214 (20.06)
**Sex**	Male	608 (56.98)
	Female	459 (43.02)
**Place of residence**	Rural	474 (44.42)
	Urban	593 (55.58)
**Marital status**	Single	474 (44.42)
	Married	509 (47.71)
	Divorced	46 (4.31)
	Widowed	38 (3.56)
**Level of education**	Illiterate	61 (5.72)
	Primary school	134 (12.56)
	Middle school	179 (16.78)
	Diploma and higher	693 (64.94)
**Income Level**	No income	381 (35.71)
**(per month)**	10–20 million Rials	549 (51.45)
	21–30 million Rials	102 (9.56)
	> 30 million Rials	35 (3.28)
**Total**		**1067 (100)**

As presented in Table 2, the total quality of nursing care from the patients' perspectives was 191.47 ± 19.51, indicating a moderate quality level: psychosocial (91.34 ± 9.34), physical (65.72 ± 10.18), and communication (34.41 ± 6.21).

**Table 2:** Frequency distribution of quality of nursing care and its dimensions from patients’ perspectives

Dimensions	Level of quality	Frequency (Percent)
Psychosocial	Low	127 (11.90)
	Moderate	748 (70.10)
	High	192 (18.00)
	**Mean ± SD**	**91.34 ± 9.34**
Physical	Low	152 (14.25)
	Moderate	716 (67.10)
	High	199 (18.65)
	**Mean ± SD**	**65.72 ± 10.18**
Communication	Low	208 (19.49)
	Moderate	663 (62.14)
	High	196 (18.37)
	**Mean ± SD**	**34.41 ± 6.21**
**Total quality of nursing care**	Low	175 (16.40)
	Moderate	684 (64.11)
	High	208 (19.49)
	**Mean ± SD**	**191.47 ± 19.51**

In Table 3, among the domains of the psychosocial dimension from the patients' perspectives, "paying attention to patients’ request to meet a clergyman" had the lowest score (2.43 ± 0.27).

**Table 3:** Frequency distribution of psychosocial domains of quality of nursing care from patients’ perspectives

Domains of psychosocial dimension	Mean** ± **SD
1. Responding to patients ‘ questions Patiently	2.66 ± 0.28
2. Providing an appropriate environment to answer patient's questions	2.73 ± 0.19
3. Talking to colleagues only about meeting patients' needs	2.76 ± 0.22
4. Tone of the nurses' voice indicating their interest in solving patients ‘ problems and meeting their needs	2.83 ± 0.27
5. Paying attention to patients ‘ words	2.71 ± 0.16
6. Feeling satisfied after talking with nurses	2.85 ± 0.18
7. Calling patients by name, not by bed number	2.93 ± 0.14
8. Introducing nurses to patients	2.91 ± 0.23
9. Nurses' rational behaviors in cases of inappropriate behaviors exhibited by patients	2.65 ± 0.28
10. Spending more time with nurses when a patient feels lonely	2.73 ± 0.36
11. Talking to patients if they are tired of treatment and encouraging them to pursue treatment	2.70 ± 0.17
12. Not getting angry or expressing impolite words when dealing with patients	2.90 ± 0.38
13. Nurses ‘ ability to diagnose and reduce patient anxiety	2.87 ± 0.14
14. Staying with patients if they feel anxious and sparing efforts to decrease such a feeling	2.85 ± 0.14
15. Allowing one of the patient's family members to stay with him/her if the anxiety level does not decrease	2.93 ± 0.29
16. Explaining once more with a happy face and without expressing discomfort to patients	2.78 ± 0.21
17. Explaining medical care procedures and tests to patients	2.83 ± 0.16
18. Informing patients about their recovery process	2.78 ± 0.19
19. Informing patients about the arrival and departure of the nurse	2.68 ± 0.29
20. Adopting therapeutic measures when they have the least interference with the appointment time	2.96 ± 0.31
21. Providing an appropriate environment for patients to communicate with their families	2.64 ± 0.33
22. Paying attention to patients' requests to meet a clergyman	2.43 ± 0.27
23. Teaching patients to perform religious duties considering their physical status	2.61 ± 0.25
24. Providing information to patients about their diseases	2.93 ± 0.11
25. Dominating trust between patients and nurses	2.80 ± 0.17
26. Paying attention to patients ‘ opinions regarding the provided care and, if possible, observing them	2.73 ± 0.26
27. Answering patients ‘ questions with kindness and patience	2.82 ± 0.28
28. Introducing a new patient to other patients	2.59 ± 0.17
29. Explaining to patients the reasons why to observe some ward rules	2.69 ± 0.32
30. Introducing a patient to patients with similar problems	2.64 ± 0.19
31. Training patients to do their personal chores alone considering their physical status	2.81 ± 0.24
32. Encouraging families to care for their patients	2.85 ± 0.18
33. Talking about topics of interest to patients during care procedures	2.76 ± 0.22
**Total**	**91.34 ± 9.34**

According to Table 4, among the physical domains, from the patients' perspectives, "using aromatic substances to deodorize the environment" had the lowest score (2.39 ± 0.29).

**Table 4:** Frequency distribution of physical domains of quality of nursing care from patients’ perspectives

Domains of the physical dimension	Mean** ± **SD
1. Observing personal hygiene by nurses	2.83 ± 0.41
2. Making necessary equipment available	2.51 ± 0.28
3. Adjusting the bar next to the bed and explaining about it	2.53 ± 0.36
4. Meeting daily health needs	2.49 ± 0.39
5. Helping patients to do personal chores in case of disability	2.63 ± 0.31
6. Monitoring environmental health daily	2.44 ± 0.22
7. Using aromatic substances to deodorize the environment	2.39 ± 0.29
8. Providing psychological support for patients	2.59 ± 0.34
9. Adopting necessary care measures with appropriate skills	2.64 ± 0.38
10. Adopting the necessary care to maintain skin health	2.62 ± 0.32
11. Paying attention to patients ‘ weight changes	2.55 ± 0.24
12. Paying attention to patients ‘ dietary pattern	2.51 ± 0.39
13. Paying attention to patients ‘ sleep and rest pattern	2.48 ± 0.25
14. Paying attention to patients ‘ defecation pattern	2.49 ± 0.34
15. Recognizing the cause of pain quickly and trying to eliminate or reduce it	2.62 ± 0.19
16. Paying attention to patients ‘ dissatisfaction with the venous injection site and trying to resolve it	2.52 ± 0.24
17. Explaining about the correct performance of sports movements, if required	2.46 ± 0.36
18. Teaching proper breathing and discharging lung secretions and its cause	2.50 ± 0.33
19. Helping patients to get out of bed and walk	2.51 ± 0.23
20. Training intermittent rest during activities to conserve energy	2.44 ± 0.26
21. Teaching reasons for getting out of bed after surgery	2.43 ± 0.37
22. Explaining reasons for following a special diet	2.53 ± 0.28
23. Asking patients ‘ names before giving medicines	2.48 ± 0.22
24. Explaining the therapeutic effects of medicines	2.47 ± 0.19
25. Explaining the side effects and warnings of medicines	2.50 ± 0.31
26. Asking about the patient's history of allergies to a particular food or medicine	2.56 ± 0.28
**Total**	**65.72 ± 10.18**

As shown in Table 5, among the communication domains from the patients' perspectives, "providing the patient's family with enough time to ask their questions" had the lowest score (2.48 ± 0.26).

**Table 5:** Frequency distribution of communication domains of quality of nursing care from patients’ perspectives

Domains of communication dimension	Mean** ± **SD
1. Sharing (patient) feelings with nurses easily	2.76 ± 0.32
2. Ask nurses questions about the disease easily	2.66 ± 0.45
3. Listening well to patients ‘ words	2.61 ± 0.36
4. Providing the patient's family with enough time to ask their questions	2.48 ± 0.26
5. Family satisfaction with nurses' responses	2.53 ± 0.39
6. Understanding the anxiety of the patient's family and providing the necessary training to reduce their anxiety	2.55 ± 0.25
7. Informing the patient's family about the patient's recovery process	2.51 ± 0.22
8. Ensuring patients of the confidentiality of their secrets	2.84 ± 0.29
9. Predicting some of the patient's needs even before being requested by the patient	2.63 ± 0.41
10. Establishing appropriate communication between nurses and other medical staff	2.87 ± 0.30
11. Introducing necessary referral resources and organizations to patients	2.58 ± 0.34
12. Satisfying patient's needs in a calm and anxiety-free environment	2.60 ± 0.37
13. Paying attention to patients ‘ needs while talking to them	2.79 ± 0.26
**Total**	**34.41 ± 6.21**

Findings indicated a positive relationship between age nurses’ services quality (*r* = 0.536, *P* = 0.03), signifying that with increasing age, patients showed better views regarding nurses’ services quality. The perceived nurses service quality was subject to sex (*P* = 0.01, t = 1.921) and place of residence (*P* = 0.02, t = 1.873). The average quality of nursing care was higher from the perspectives of female patients (193.29 ± 19.87) than male patients (189.66 ± 18.65). Moreover, patients living in rural areas (197.76 ± 20.34) had a higher average nursing care quality than urban residents (185.19 ± 16.24).

There was no statistically significant difference in the quality of nursing care according to marital status, education level, and income level (Table 6).

**Table 6:** Relationship between the quality of nursing care from patients’ perspectives and their demographic specifications

**The main research variable**	**Demographic specifications**	**Category**	**Mean ± SD** **quality of nursing care**	**Type of test and significance**	
				Pearson correlation coefficient	*P*-Value*
**Quality of** **nursing care**	Age	< 20	189.56 ± 16.88	0.536	**0.03**
		20–35	190.49 ± 17.62		
		36–50	192.64 ± 19.31		
		> 50	193.19 ± 20.11		
	--			t-test (t)	*P*-Value*
	Sex	Male	189.66 ± 18.65	1.921	**0.01**
		Female	193.29 ± 19.87		
	Place of residence	Rural	197.76 ± 20.34	1.873	**0.02**
		Urban	185.19 ± 16.24		
	--			ANOVA (F)	*P*-Value*
	Marital status	Single	196.43 ± 20.28	1.501	0.11
		Married	192.49 ± 19.76		
		Divorced	187.61 ± 17.84		
		Widowed	189.36 ± 18.47		
	Level of education	Illiterate	193.64 ± 19.29	1.224	0.10
		Primary school	195.89 ± 20.14		
		Middle school	189.71 ± 18.57		
		Diploma and higher	186.63 ± 17.88		
	Income level	No income	194.87 ± 19.18	1.144	0.16
		10–20 million Rials	196.59±20.32		
		21–30 million Rials	188.57 ± 18.35		
		> 30 million Rials	185.86 ± 17.72		

^*^ *P*-Value; Correlation is significant at the 0.05 level

## Discussion

The aim of this study was to investigate patients' perceptions of the quality of nursing services in teaching hospitals settings. According to the first part of the research results, the average quality of nursing care from patients' perspectives was assessed to be moderate. In line with the findings of this study, the results of several studies indicate the average level of quality of nursing services from the perspective of patients [38–45]. However, the findings of some studies showed that the quality of nursing services from the patients' point of view was at a desirable and acceptable level [46–55]. The results of some other studies also indicated an inappropriate and unfavorable level of quality of nursing services [56, 57]. The difference between the results of this study and other similar ones could be due to the research environment, society and the sample size, and differences in the socio-cultural status of the participants. In the present study, the prevalence of the Covid19 pandemic could affect the quality of nursing services and subsequently patients' perception of this quality, leading to reducing the quality of care to a moderate level. Therefore, it is necessary for senior managers of hospitals to plan and take action to improve the quality of nursing services and positive perceptions of patients towards this quality.

The average quality of nursing care in the psychosocial dimension from the patients’ perspectives was assessed to be at a moderate level. In this regard, the results of various studies indicate different levels of quality of nursing care in the psychosocial dimension. In the study of Dabirian and colleagues, most of the patients assessed the quality of nursing care in the psychosocial dimension as poor [38]. However, according to Neishabory et al., the quality of nursing care in the psychosocial dimension was not desirable for patients [32]. Haghighi Khoshkho et al. also reported the unsatisfactory quality of care for most patients in psychosocial and communication dimensions [35]. In Zamanzadeh et al.’s study regarding the patients' attitudes towards the quality of care, the highest dissatisfaction was associated with meeting patients’ social needs [57]. Also, in the study of Jamsahar et al., less than half of the patients (37.4%) reported the quality of nursing services in the desired psychosocial dimension [58]. The results of another study showed that hospitalized patients expect psychosocial interventions from health professionals to reduce mental health risks [59]. The findings of the present study and the above studies indicate that the psychosocial dimension of service quality needs more attention from nurses. Nurses have a legal and ethical responsibility and commitment to the quality of care they provide and should know that their psychosocial skills and expertise and skills in providing care affect the patient's perception of the quality of care. Since the nurse's primary task is to meet patients’ basic needs by communicating, intervening, assisting, and supporting their treatment, if nurses can communicate properly with patients, the quality of nursing care will be promoted. Moreover, if the care provided is appropriate and accurate, patients will be more satisfied [60].

Regarding another part of the findings of the present study, the average quality of nursing care in the physical dimension was evaluated by patients to be at a moderate level. In Haghighi Khoshkho et al.'s study, 42% of patients rated the quality of nursing care in the physical dimension as acceptable [35]. Hosseinzadeh et al. also revealed that nurses' caring behaviors were acceptable and that they focused more on the physical dimension of care [61]. In the study of Jamsahar et al., Only 38.8% of patients reported a good quality of nursing care in the physical dimension [58]. Gishu et al. further reported that the quality of nursing care in the physical dimension was below average [44].

Studies in developing countries have highlighted the physical dimensions of care more than the psycho-emotional dimensions [62, 63]. It is important to note that nurses should value physical and psychosocial care equally when working with patients [63, 64]. According to patients, a good nurse is someone who, in addition to providing acceptable care, can provide appropriate general and physical support [64]. Since behaviors in the physical dimension are more tangible and measurable than those in the psychosocial dimension, nurses may prefer to focus on the caring behaviors that are most frequently questioned [65]. Therefore, pay attention to the physical aspect of the quality of nursing care, such as observing personal hygiene by nurses, helping patients to do personal chores in case of disability, recognizing the cause of pain quickly and trying to eliminate or reduce it, using aromatic substances to deodorize the environment, etc. by nurses and nursing managers seems necessary.

Another dimension was the quality of nursing care in communication. From the patients' perspectives in this study, the quality of nursing care in communication was between moderate levels. Neishabory et al. declared that the quality of nursing care in the communication dimension was acceptable from the perspective of 24.7% of patients [32]. Haghighi Khoshkho et al. revealed that most patients reported low quality of care in the communication dimension [35]. In addition, in the study of Jamsahar et al., 41.3% of nurses reported the quality of nursing care in the communication dimension as desirable [58]. In this regard, Fallowfield and Jenkins emphasize that the effective and useful relationship of the nurse with the patient leads to a level of recovery, pain control and adherence to treatment regimens and improve the patient's mental and psychological function [66]. The Joint Accreditation Commission of Health Care Institutions also found that poor communication could affect patient's safety and satisfaction with the quality of care [67].

Since nurses’ primary task is to meet the patient's basic needs by communicating, intervening, helping, and assisting in treatment, the quality of nursing care will increase if they can communicate properly with patients, and patients will be more satisfied if the provided care is appropriate and accurate [32]. Moreover, promoting communication skills increases the quality of services in communication dimensions. In general, not understanding patients’ needs and desires by the nursing staff can be considered as the most important factor affecting nurses’ non-response. By promoting nurses' communication skills, especially the art of listening and inducing a sense of human dignity, it is possible to increase nurses' understanding of patients' needs and wants [56].

Findings confirmed a positive relationship between age and nurses services quality, indicating that with increasing the age, patients showed better view of nurses services quality. The perceived quality of nursing services was subject to patients’ sex and place of residence. In this regard, the findings of a study by Lee et al. in Canada conducted on 1866 patients showed that the age variable was significantly associated with their views on the quality of services [68]. Akin and Erdogan's study in Turkey also found that there was a statistically significant relationship between patients’ satisfaction with nursing care and patients' age and sex, so that older patients and female patients were more satisfied with the quality of nursing services. [69]. Adam et al. Also reported a statistically significant difference in the quality of nursing services based on patients' age [70].

Regarding the sex variable, in the studies of Abbasi Farajzadeh et al. [49] and EI- Nagger et al., [71] the level of satisfaction with nursing care was higher in male patients than female patients. The results of Taghavi Larijani and Najafi's research showed that patients' satisfaction with nursing services was different according to sex [72]. The difference in views between female and male patients may be due to the female-male relationship or behavioral differences between the sexes. The high average score of quality of nursing services from the perspective of women compared to men may be related to their emotional personality and maternal role. Women generally pay more attention to intangible aspects of services provided by nurses and percept the nursing services mostly from the communication and psychological aspects.

According to the findings of the present study, the study of Woldeyohanes et al. showed a significant difference in the quality of nursing services from the patients' point of view according to their location [73], so that patients living in rural areas had higher satisfaction with the quality of nursing services. It seems that one of the reasons for this result could be cultural and social differences and lower expectations of patients living in rural areas. It also seems that due to the lack of access to health services in rural areas compared to urban residents in Iran, when rural residents receive medical services from nurses, they would manifest much more positive feedback as opposed to those who receive such services frequently in rural areas. In addition, urban residents commonly benefit more from health services and are more oriented about how to receive such services, leading to becoming more sensitive to the services they receive; all would place them in a situation with high expectations toward the quality of the nursing services that they may need.

Based on the findings of this study, the quality of nursing services from the perspective of patients with primary school education and income of 10–20 million Rials (380.735–764.47 US $), as well as single patients, was better compared to their peers, but this difference was not statistically significant. Consistent with the present study, in Akin and Erdogan's study in Turkey [69] and Alhusban and Abualrub's study in Jordan [74], there was no statistically significant relationship between nursing care satisfaction and patients' education level. However, in the study of Lee et al., the satisfaction of patients with higher education was lower than that of patients with lower education [68].

Various studies have considered the level of education of patients as an influential factor on their view of the quality of nursing services [40, 75–77]. Regarding the income variable, the results of studies by Abbasi Farajzadeh et al. [49] and Tavasoli et al. [78] showed that there is an inverse relationship between patients’ income level and their satisfaction with nursing care; hence, that with increasing patients' monthly income, their level of satisfaction with nursing care decreased. People with higher education and income seem to have lower satisfaction and lower quality of nursing services for reasons such as higher expectations of the health care system, more social communication, better access to information resources, and greater ability to identify system deficiencies. Contrary to the findings of this study, Garroutte and Robert in their study reported a statistically significant relationship between patients' satisfaction with the quality of nursing services based on marital status [79] that one of the reasons for this difference could be different in the sample.

## Conclusion

According to the findings, the quality of nurses 'care was evaluated from the patients' perspectives at a moderate level. Accordingly, nursing managers and hospital officials are recommended to pay attention to how nursing care is provided as well as the quality of the offered services. It is also necessary to hold courses and workshops with an emphasis on promoting nurses' communication skills to promote the quality of nursing care. Further attention should also be paid to the observance of accreditation standards to provide care services to patients by nurses. Due to their different backgrounds and different experiences, patients and nurses have different perceptions and perspectives on the quality of care services. Accordingly, it is necessary to re-evaluate the quality care standards using a client-centered approach to take measures to increase the appropriate relationship between patients and nurses and consider patients’ psychosocial needs. This shortens the length of the patient's hospital stay and reduces the costs imposed on the treatment system.

One of the limitations of this study was that only the patients’ views and opinions were examined and nurses’ perspectives were not assessed. In contrast, one of the strengths of this study was the large sample size of the study.

## Data Availability

All the data is presented as a part of tables or figures. Additional data can be requested from the corresponding author.

## References

[1] Androniceanu A, Sabie OM, Pegulescu A (2020). An integrated approach of the human resources motivation and the quality of health services. Theoretical and Empirical Researches in Urban Management.

[2] Raadabadi M, Bahadori M, Ravangard R, Mousavi SM (2017). Comparing the quality of nursing services between two public and private hospitals. International Journal of Healthcare Management.

[3] Barati O, Najibi M, Yusefi AR, Dehghan H, Delavari S (2019). Outsourcing in Shiraz University of Medical Sciences; a before and after study. J Egypt Public Health Assoc.

[4] Ryu JI, Kim K (2018). The influence of nursing care integration services on nurses’ work satisfaction and quality of nursing care. J Nurs Manag.

[5] Sagong H, Lee GE (2016). Person-centered care and nursing service quality of nurses in long-term care hospitals. J Korean Acad Community Health Nurs.

[6] Nadi A, Shojaee J, Abedi G, Siamian H, Abedini E, Rostami F (2016). Patients’ expectations and perceptions of service quality in the selected hospitals. Medical Archives.

[7] Gupta KS, Rokade V (2016). Importance of quality in health care sector: A review. J Health Manag.

[8] Bowers J, Cheyne H (2016). Reducing the length of postnatal hospital stay: implications for cost and quality of care. BMC Health Serv Res.

[9] Vanholder R, Annemans L, Brown E, Gansevoort R, Gout-Zwart JJ, Lameire N (2017). Reducing the costs of chronic kidney disease while delivering quality health care: a call to action. Nat Rev Nephrol.

[10] Nugraheni R, Kirana GR (2018). The analysis quality of service and patient satisfaction participants of health BPJS in interior services in hospital X of Kediri City. Journal of Global Research in Public Health.

[11] Armstrong G (2019). Quality and safety education for nurses teamwork and collaboration competency: Empowering nurses. The Journal of Continuing Education in Nursing.

[12] Lee MJ, Yoon SH, Cho YC (2016). Relationship between psychosocial factors, job stress contents, fatigue symptoms and quality of nursing services among general hospital nurses. Journal of the Korea Academia-Industrial cooperation Society.

[13] Nikmanesh P, Mohammadzadeh B, Nobakht S, Yusefi AR (2018). Nurses Communication Skills Training and Its Effect on Patients’ Satisfaction in Teaching Hospitals of Shiraz University of Medical Sciences. Iran J Health Sci.

[14] Nomura AT, Pruinelli L, Da Silva MB, Fátima Lucena A, Abreu Almeida M (2018). Quality of electronic nursing records: the impact of educational interventions during a hospital accreditation process. CIN: Comp Informa Nur.

[15] Bayraktar AK, Sivrikaya SK (2018). Nursing Ethics in the Emergency Department/Acil Servis Hemsirelik Hizmetlerinde Etik. Journal of Education and Research in Nursing.

[16] Karaca A, Durna Z (2019). Patient satisfaction with the quality of nursing care. Nurs Open.

[17] Ryan C, Powlesland J, Phillips C, Raszewski R, Johnson A, Banks-Enorense K (2017). Nurses' perceptions of quality care. J Nurs Care Qual.

[18] Ndengeyingoma A, Ruel J (2016). Nurses’ representations of caring for intellectually disabled patients and perceived needs to ensure quality care. J Clin Nurs.

[19] Schroyer CC, Zellers R, Abraham S (2016). Increasing registered nurse retention using mentors in critical care services. Health News.

[20] Barkhordari-Sharifabad M, Ashktorab T, Atashzadeh-Shoorideh F (2018). Ethical leadership outcomes in nursing: A qualitative study. Nurs Ethics.

[21] Nantsupawat A, Nantsupawat R, Kunaviktikul W, Turale S, Poghosyan L (2016). Nurse burnout, nurse-reported quality of care, and patient outcomes in Thai hospitals. J Nurs Scholarsh.

[22] Bakhteari Z, Hanifi N, Amini K, Jafari VN (2018). Quality of Nursing Services in Dialysis Center of Valiasr Hospital in Zanjan from Nurses and Patients' Viewpoint Using the SERVQUAL Model. Iran J Nurs.

[23] Mollaoğlu M, Çelik P (2016). Evaluation of emergency department nursing services and patient satisfaction of services. J Clin Nurs.

[24] Mensa M, Taye A, Katene S, Abera F, Ochare O (2017). Determinants of patient satisfaction towards inpatient nursing services and its associated factors in, Gamo Gofa zone, SNNPR, Ethiopia, April 2017. MOJ Clin Med Case Rep.

[25] Peyrovi H, Bahadori A, Ashghali- Farahani M, Haghani H (2013). Comparison of inpatients' satisfaction with different domains of nursing care. Quarterly Journal of Nursing Management.

[26] Aiken LH, Sermeus W, Van den Heede K, Sloane DM, Busse R, McKee M (2012). Patient safety, satisfaction, and quality of hospital care: cross sectional surveys of nurses and patients in 12 countries in Europe and the United States. BMJ.

[27] Vogus TJ, McClelland LE (2016). When the customer is the patient: Lessons from healthcare research on patient satisfaction and service quality ratings. Hum Resour Manag Rev.

[28] Elewa A, Elkattan BA (2017). Effect of an educational program on improving quality of nursing care of patients with thalassemia major as regards blood transfusion. American Journal of Nursing Research.

[29] Lu SJ, Kao HO, Chang BL, Gong SI, Liu SM, Ku SC (2020). Identification of quality gaps in healthcare services using the SERVQUAL instrument and importance-performance analysis in medical intensive care: a prospective study at a medical center in Taiwan. BMC Health Serv Res.

[30] Karaca A, Durna Z (2019). Patient satisfaction with the quality of nursing care. Nurs Open.

[31] MohajjelAghdam A, Hassankhani H, Zamanzadeh H, Khameneh S, Moghaddam S (2013). Nurses' Performance on Iranian Nursing Code of Ethics from Patients' Perspective. Iran J Nurs.

[32] Neishabory M, Raeisdana N, Ghorbani R, Sadeghi T (2010). Nurses' and patients' viewpoints regarding quality of nursing care in the teaching hospitals of Semnan University of Medical Sciences, 2009. Koomesh.

[33] Shannon SE, Mitchell PH, Cian KC (2002). Patients, Nurses, and physicians have differing views of quality of critical care. J Nur Schol.

[34] Sheikhi MR, Javad A (2004). Patients’ satisfaction of medical services in Qazvin educational hospitals. J Qazvin Univ Med Sci.

[35] Haghighi Khoshkho N (2004). The quality of nursing care from nurses and patients viewpoints in the Teaching hospitals of Tabriz university of Medical Sciences. [MA thesis].

[36] Fatehi R, Nezal A, Motalebi A (2019). Nurses'and elderly’s'viewpoints regarding quality of nursing care in the educational hospitals of sanandaj city. The J Urmia Nurs Midwifery Fac.

[37] Gholjeh M, Dastoorpour M, Ghasemi A (2015). The Relationship between Nursing Care Quality and Patients Satisfaction among Hospitals affiliated to Zahedan University of Medical Sciences in 2014. Jorjani Biomed J.

[38] Dabirian A, Zolfaghari H, Saidi ZA, Alavi-Majd H (2008). Views of AIDS patients regarding nursing care quality in healthcare centers affiliated to Shaheed Beheshti and Tehran Universities of Medical Sciences. Advances in Nursing & Midwifery.

[39] Nabili A, Bastani F (2020). Evaluate quality of nursing care from the viewpoint of elderly patients under hemodialysis in selected Medical Education Centers of Iran University of Medical Sciences, 2018. Iran J Nurs Res.

[40] Joolaee S, Hajibabaee F, Jalal EJ, Bahrani N (2011). Assessment of Patient Satisfaction from Nursing Care in Hospitals of Iran University of Medical Sciences. HAYAT.

[41] Hajian K (2007). Satisfaction of hospitalized patients of health care services in Shahid Beheshti and Yahyanezhad hospitals of Babol. Journal of Babol University of Medical Sciences.

[42] Gholjeh M, Ghaljaee F, Mazloom A (2008). Correlation between nurses' practice ability and patient satisfaction of nursing care. Pub Shahid Beheshti School of Nursing and Midwifery.

[43] Chao M, Shih CT, Hsu SF (2016). Nurse occupational burnout and patient-rated quality of care: The boundary conditions of emotional intelligence and demographic profiles. Jpn J Nurs Sci.

[44] Gishu T, Weldetsadik AY, Tekleab AM (2019). Patients’ perception of quality of nursing care; a tertiary center experience from Ethiopia. BMC Nurs.

[45] Mrayyan MT (2006). Jordanian nurses’ job satisfaction, patients’ satisfaction and quality of nursing care. Int Nurs Rev.

[46] Khaki S, Esmaeilpourzanjani S, Mashouf S (2018). Nursing cares quality in nurses. Scientific Journal of Nursing, Midwifery and Paramedical Faculty.

[47] Dehghani A, Ordoubadi N, Shamsizadeh M, AM Nasab P, Talebi M (2014). Perspective of patients about compliance with standards of professional ethics in nursing practice. J nur educ.

[48] Miri K, Keshavarz A, Shirdelzadeh S, Parsa P (2015). The relationship between nurses' spiritual intelligence and quality of nursing care based on nurses'& patients' viewpoints. J Urmia Nurs Midwifery Fac.

[49] AbbasiFarajzadeh M, Vahedian-Azimi A, MirJavadi SA, Karimi L (2019). Survey of the Patients' Satisfaction with Services provided in an Iranian Naval Hospital in 2019: A Cross-sectional Study. J Mar Med.

[50] Alasad J, Tabar NA, AbuRuz ME (2015). Patient satisfaction with nursing care. J Nurs Adm.

[51] Mogadasiyan S, Abdolahzadeh F, Rahmani A, Nikanafar A, Firoziyan A (2013). Satisfaction with nursing care and related factors in hospitalized cancer patients in Shahid Ghazi hospital in Tabriz. Journal of Urmia Nursing & Midwifery Faculty.

[52] Mudallal RH, Saleh MY, Al-Modallal HM, Abdel-Rahman RY (2017). Quality of nursing care: the influence of work conditions and burnout. International Journal of Africa Nursing Sciences.

[53] Ríos-Risquez MI, García-Izquierdo M (2016). Patient satisfaction, stress and burnout in nursing personnel in emergency departments: A cross-sectional study. Int J Nurs Stud.

[54] Tornvall E, Wilhelmsson S (2010). Quality of nursing care from the perspective of patients with leg ulcers. J Wound Care.

[55] Grondahl W, Muurinen H, Katajisto J, Suhonen R, Leino-Kilpi H (2019). Perceived quality of nursing care and patient education: a cross-sectional study of hospitalised surgical patients in Finland. BMJ Open.

[56] Ebrahimian T, Salehi A, Rejalian F, Jabbari A (2017). Evaluation of the nursing services quality from the patient perspective by using the Kano and SERVQUAL combines model. Health Research Journal.

[57] Zamanzadeh V, Moghadasiyan S, Vali Zadeh L, Haghighi KN (2004). Comparing the patients and nurses viewpoints about the nursing care quality Offered in educational hospital. Journal of Nursing and Midwifery of Tabriz Medical Sciences University.

[58] Jamsahar M, Khaki S, EsmeilpourZanjani S, Mashouf S (2020). Comparison of quality of nursing cares from the perspective of nurses and patients. Scientific Journal of Nursing, Midwifery and Paramedical Faculty.

[59] Lipp MEN, Frare A, Santos FU (2007). Efeitos de variáveis psicológicas na reatividade cardiovascular em momentos de stress emocional. Estud Psicol.

[60] Hylen U, Kjellin L, Pelto-Piri V, Warg LE (2018). Psychosocial work environment within psychiatric inpatient care in Sweden: Violence, stress, and value incongruence among nursing staff. Int J Ment Health Nurs.

[61] Hosseinzadeh H, Mohammadi M, Shamshiri M (2019). The study of caring behaviors and its determinant factors from the perspective of nurses in educational hospitals of Ardabil. Journal of Health and Care.

[62] Li YS, Yu WP, Yang BH, Liu CF (2016). A comparison of the caring behaviours of nursing students and registered nurses: implications for nursing education. J Clin Nurs.

[63] Hasson H, Arnetz JE (2008). Nursing staff competence, work strain, stress and satisfaction in elderly care: a comparison of home-based care and nursing homes. J Clin Nurs.

[64] Salimi S, Azimpour A, Fesharaki M, Mohammadzadeh S (2012). Nurses' Perception of Importance of Caring Behaviors and Its Determinant factors. Journal of Urmia Nursing & Midwifery Faculty.

[65] Hajinezhad M, Rafii F, Jafarjalal E, Haghani H (2007). Relationship between nurse caring behaviors from patients’ perspectives & their satisfaction. Iran J Nurs.

[66] Fallowfield L, Jenkins V (1999). Effective communication skills are the key to good cancer care. Eur J Cancer.

[67] Hamdan-Mansour AM, Aboshaiqah AE, Thultheen IN, Salim WM, Azzeghaiby SN, Anani MA (2014). Patients’ satisfaction about nurses’ competency in practicing communication skills. Life Science Journal.

[68] Lee DS, Tu JV, Chong A, Alter DA (2008). Patient satisfaction and its relationship with quality and outcomes of care after acute myocardial infarction. Circulation.

[69] Akin S, Erdogan S (2007). The Turkish version of the Newcastle Satisfaction with Nursing Care Scale used on medical and surgical patients. J Clin Nurs.

[70] Adam C, Patiraki E, Lemonidou C, Radwin LE, Charalambous A, Charalambous M (2017). Quality of nursing care as perceived by cancer patients: a cross-sectional survey in four European countries. J BUON.

[71] El-Nagger NS, Ahmed S, Elsayed LA, Khamis H (2013). Patients' satisfaction regarding nursing care provided in different hospitals in Makkah AL Mukramah. Life science journal.

[72] TaghaviLarijani T, Najafi F (2019). Patient Satisfaction Survey of Nursing Care and Services in Iran: A systematic review. J Educ Ethics Nurs.

[73] Woldeyohanes TR, Woldehaimanot TE, Kerie MW, Mengistie MA, Yesuf EA (2015). Perceived patient satisfaction with in-patient services at Jimma University Specialized Hospital. Southwest Ethiopia BMC research notes.

[74] Alhusban MA, Abualrub RF (2009). Patient satisfaction with nursing care in Jordan. J Nurs Manag.

[75] Farzianpour F, Hosseini SM, Hosseini SS, MovahedKor E, Amerzadeh M (2013). The Relationship Between Nursing Managers' Self-Reliance And Patients' Satisfaction In Hospitals Affiliated To Tehran University Of Medical Sciences (TUMS). Payavard.

[76] Findik UY, Unsar S, Sut N (2010). Patient satisfaction with nursing care and its relationship with patient characteristics. Nurs Health Sci.

[77] Quintana JM, González N, Bilbao A, Aizpuru F, Escobar A, Esteban C (2006). Predictors of patient satisfaction with hospital health care. BMC Health Serv Res.

[78] Tavasoli S, Alhani F (2011). Evaluation of parental satisfaction of nursing care in thalassemic children. Journal of Urmia Nursing & Midwifery Faculty.

[79] Garroutte E, Robert M (2004). Patient satisfaction and ethane identity among American Indian older adults. Am J Psych.

